# Development of a suite of short planetary health learning resources by students for students as future health professionals

**DOI:** 10.3389/fmed.2024.1439392

**Published:** 2024-09-04

**Authors:** Catarina Pais Rodrigues, Evangelos Papageorgiou, Michelle McLean

**Affiliations:** ^1^National School of Public Health, Lisbon, Portugal; ^2^Medical Student Alliance for Global Education (MeSAGE)-ScholarRx, Louisville, KY, United States; ^3^Evangelismos Hospital, Athens, Greece; ^4^Faculty of Health Sciences and Medicine, Bond University, Gold Coast, QLD, Australia

**Keywords:** health professions education, planetary health, meaningful student involvement, open education, sustainable health education

## Abstract

Planetary health recognizes the interdependencies between human health and the well-being of the Earth’s ecosystems. Human activities have led to the disruption and transformation of natural systems and a range of global environmental changes such as climate change, air pollution, and biodiversity loss. Health professionals must be equipped to deal with the health impacts of global environmental changes. This article describes the development and usage of a suite of 14 short online learning resources (‘bricks’) on Planetary Health on the ScholarRx platform. There are several principles that inform the development of these bricks, including learner-centric, peer learning, diversity, equity and inclusion, and authentic learning. The content is developed using a student-educator collaboration model, supported by an editorial team. The suite of 14 modules was published in June 2023, with the initial usage data promising with 1,990 views in the first 10 months. These digital, modular resources allow for easy dissemination and can be incorporated in different programs depending on context and need.

## 1 Introduction

In 2009, Costello and colleagues identified climate change as potentially the greatest threat to global health in the 21^st^ century, concluding that this *“raises many challenging and urgent questions for politicians, civil servants, academics, health professionals, NGOs, pressure groups and local communities”* (p. 1728) (1). Despite extensive deliberation, meetings, and conferences, inaction combined with a growing human population, resource extraction, fossil fuel burning, deforestation and pollution have led to the transformation and disruption of many of Earth’s systems. The planet’s inhabitants now face a triple planetary crisis: A changing climate, biodiversity loss, and pollution (2).

Health and well-being are inextricably linked to the health of the planet. Changing land and sea temperatures have led to droughts, storms, floods, heatwaves, and rising sea levels, resulting in the loss of human life and property (3). These same weather patterns are also having devastating impacts on plants and animals, displacing many species, disrupting breeding cycles, and causing death (4). It is not surprising that in late 2023, health journal editors declared that it was time to consider the climate and nature crises as one urgent global health emergency (5). Our natural environment, which provides us with our basic needs of air, food, and water, as well as a range of other ‘services,’ is now the foremost determinant of our health. Air pollution is responsible for 9 million premature deaths annually (6), with increasing evidence of links to other conditions such as Alzheimer’s disease (7). The millions of tonnes of plastics that pollute every ecosystem, including the deepest oceans, have introduced micro- and nano-plastics (MNPs) throughout our food chains (8). These MNPs are recognized as endocrine disruptors and carcinogens, as are the ‘forever’ chemicals, per- and poly-fluoroalkyl substances (PFAS) (9).

Health professionals must be equipped to deal with the impacts of the triple planetary crisis on patients, communities, and the health system. Increasingly, health professionals are being called on to advocates for vulnerable populations—such as children, pregnant women, and the elderly—as well as for the planet (10). Health professions education (HPE) has, however, been slow to include concepts such as environmental and ecological determinants of health, environmentally sustainable healthcare, and climate and ecological justice in curricula. A 2020 International Federation of Medical Students’ Associations (IFMSA) survey of 2,817 medical schools in 112 countries among found that only 15% had included climate change and health in the curriculum, and only 11% had included air pollution and health (11). It is not surprising therefore that some medical students have become educational activists, working to ensure that they are prepared for a just and sustainable future for all (12–14).

While initial calls to action in HPE focused on sustainable healthcare to reduce carbon emissions and mitigate climate change (15, 16), there is now a growing recognition of the need for a more holistic approach that includes the health of the planet. Early efforts included incorporating ‘sustainability’ into the UK’s Graduate Outcomes Statements (e.g., GMC Good Doctor 2018) and addressing the impacts of climate change (12). However, this focus on carbon emissions is increasingly seen as too narrow. A broader perspective that considers issues such as resource scarcity, overconsumption, ecotoxicity, and biodiversity loss is essential (17). The Planetary Health Alliance’s Planetary Health Educational Framework, which centres the connection with Nature (18), and the Association for Medical Education in Europe’s 2021 Consensus Statement for Planetary Health and Education for Sustainable Healthcare (19)—which outlined the knowledge, skills, values, and attributes for future health professionals—were forerunners in advocating for the integration of Planetary Health in health professions education. Additionally, McKimm and McLean’s (20) article on eco-ethical leadership outlined what was needed to shift the paradigm in health professions education.

Calls for and action toward the integration of Planetary Health in health professions education are gaining momentum (21). While much of this integration has occurred at the subject (22) or program level (23, 24), other institutions have adopted these changes at the faculty or university level (25). At least one country, Australia, now has accreditation standards that include outcomes relating to climate change, sustainable development, environmental determinants of health, Planetary Health, and sustainability (26).

This article describes the development and usage of a suite of 14 Planetary Health learning resources created by the Medical Student Alliance for Global Education (MeSAGE), an initiative supported by ScholarRx. ScholarRx is a digital education platform with a vision to create a shared global curriculum system, enabling learners, educators, and institutions to create curriculum components (27). These learning resources, called ‘bricks’, comprise short, individual digital learning topics featuring narrative text and interactive multimedia that students can complete in 15 to 20 min. MeSAGE is an alliance of 11 international student organizations that is developing open educational resources, in the form of bricks, on topics that student organizations have been advocating for and deem relevant for HPE (28). Topics are prioritized based on a needs assessment completed by representatives of international student organizations. MeSAGE has published content on different topics such as Sexual and Reproductive Health and Rights, Digital Health and, in this case, Planetary Health.

Initially, the needs assessment identified climate change as the primary topic. After consultation with experts in 2022, it was acknowledged that climate change was only one of several global environmental changes that needed to be included in the medical curriculum. A Planetary Health lens was deemed more appropriate to address the broader range of environmental issues impacting health and well-being.

## 2 Pedagogical framework(s) pedagogical principles, competencies/standards underlying the educational activity

MeSAGE’s endeavors to develop educational resources for students, by students. Several principles inform the development of these resources namely learner-centric, peer learning, diversity, equity and inclusion. These bricks provide students and educators with just-in-time and just-for-you learning resources.

### 2.1 Learner-centric, peer learning

Collections of bricks are developed by students based on students’ perceived needs, thereby ensuring that the concepts and content are current and relevant. As student authors are recruited from across the world, the bricks capture the global student voice, ensuring that the content reflects global issues. Guided by an expert, students define the learning objectives and determine the content of the bricks. This approach promotes the learning of ideas and concepts through informal discussion, cognitive restructuring, and activation of knowledge (29).

### 2.2 Diversity, equity and inclusion (DEI)

MeSAGE aspires to cultivate diversity and inclusion in HPE. Content is developed by students from different regions of the world, in the case of the Planetary Health suite, students from 11 countries (Canada, Egypt, Greece, Honduras, India, Lebanon, Mexico, Nepal, Nigeria, Serbia, and the United States) contributed to the bricks. Additionally, a concerted effort is made to challenge assumptions about different countries and vulnerable populations by sharing examples from various regions of the world and discussing issues and contributions of specific populations. The scenario presented at the start of each brick and examples provided in the narrative reflect relevant global realities (e.g., a person moved from a rural village to a city and experienced an asthma exacerbation presumably from air pollution), or in instances when it is important to identify a specific region or country, the brick usually includes a perspective from a high-income country and a low-income country (e.g., wildfires in Australia and flooding in Pakistan). Furthermore, in specific bricks such as the *Introduction to Planetary Health* and the *Environmental Justice* brick, the contributions of industrialized nations to ecosystem disruption and the vulnerabilities of Global South countries and frontline states are discussed, highlighting how low-income communities and Indigenous populations are disproportionately affected by global environmental changes. All content is written using person-centered language, following the American Medical Association guide (30).

### 2.3 Brick format: a template for authentic learning

As has been described, content is delivered in the form of bricks, which are digital learning resources featuring narrative text and interactive multimedia with self-assessment items (31). These bricks follow an instructional design template designed according to well-established educational theories and practices:

#### 2.3.1 Just-in-time, just-for-you

Two of the principles identified for effective continuing medical education are convenience (just-in-time) and individualization (just-for-you) (32). Bricks allow for that convenience as they are easily accessible online are short, concise resources that should take the learner 15–20 min to complete. The bricks format also allow for learner customization, as they can choose the order in which they engage with the bricks, depending on their individual needs. As student authors, who are not experts on the subject, develop the content the bricks will also reflect the unique perspective of the target audience.

#### 2.3.2 Andragogy

Andragogy, or adult learning theory, is often introduced in juxtaposition to pedagogy, which focuses on how children learn. It emphasizes that adult learning is self-directed, relevant, and centered around a task or problem (33). Thus, each brick starts by clearly stating the learning objectives and introducing a scenario, referred to as a ‘case connection’, that the learner revisits and resolves by the end of the brick. Each brick is a discrete learning experience, allowing the learner to choose their learning pathway, whether following the sequence presented or based on their own needs or curiosity. The reference list at the end of each brick includes the most relevant publications for further reading.

#### 2.3.3 Relevance and application of knowledge

The ‘case connection’ that starts and concludes each brick depicts a real-life scenario that a HPE student might encounter, highlighting the relevance of the content for the learner. In the Planetary Health suite, these ‘case connections’ cover global experiences of the planetary crisis, from flooding to wildfires. The use of a real-life scenario promotes authentic learning, ensuring knowledge covered in the brick is transferable to real-world practice (34).

#### 2.3.4 Learning by chunking, plus a Socratic approach

Each brick is divided into sections (chunks of information). Each section begins with a question and covers a specific learning objective. The goal is to set a pace, walking learners through the content and prompting them to engage and reflect on the question posed in the heading of each section.

#### 2.3.5 Multimedia learning

Recognizing that individuals learn best through a combination of words and pictures, each brick has a minimum of two images that highlight essential material, and which are integrated in the text (35).

#### 2.3.6 Continuous assessment, with feedback

Each brick includes interactive self-assessment items after sections or paragraphs for students to evaluate their understanding of the learning objectives (36). These self-assessment items include flash cards with open-ended and short-answer questions while engaging with the brick content, plus 4–5 multiple-choice, single-best answer questions at the end. Learners can only access the answers after engaging with the interactive elements, ensuring active participation and reflection.

### 2.4 Meaningful student engagement in the development of peer learning resources

**Figure 1:**
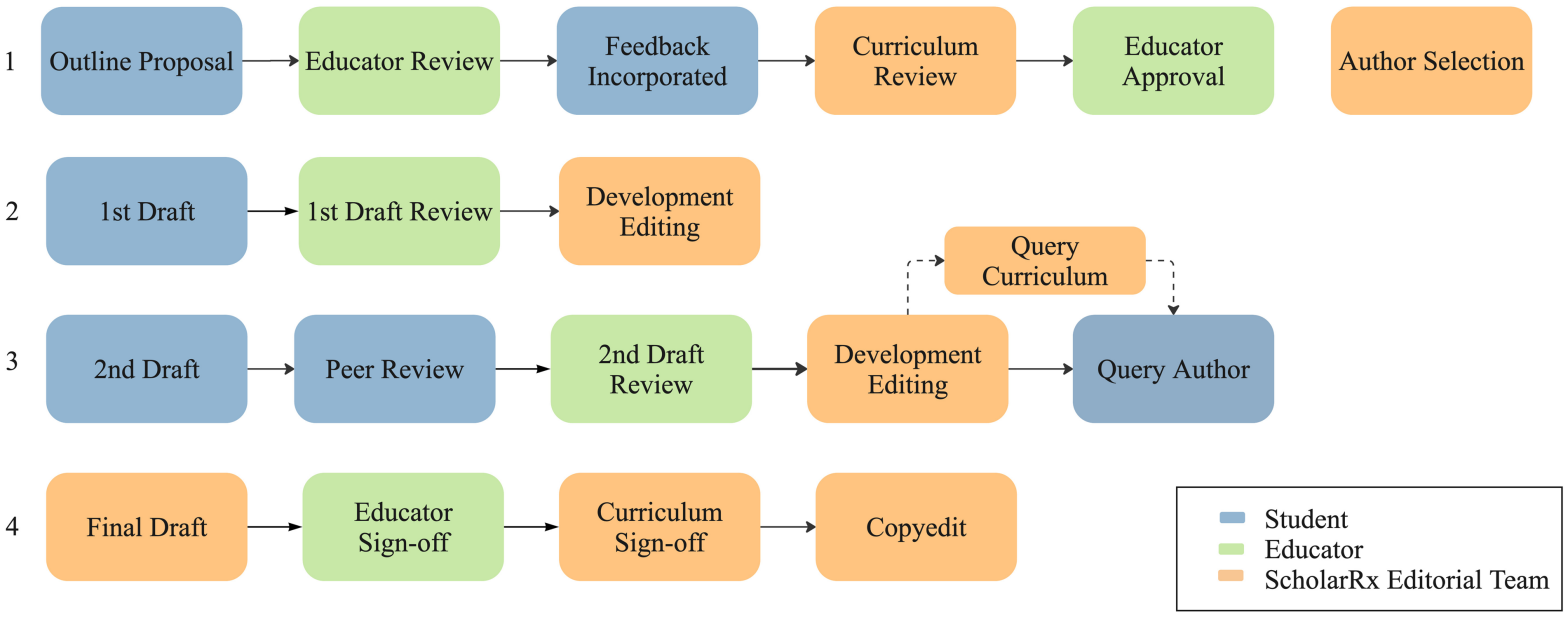
Bricks workflow.

A foundational principle of MeSAGE is meaningful student engagement in the development of educational resources through student-educator collaboration (28). The ScholarRx editorial team comprising a project manager, a curriculum manager, developmental editors and illustrators supports this collaboration, liasoning with students and the educator, editing content for clarity and consistency across modules, and creating artwork for the content. Figure 1 provides an overview of the workflow in developing a suite of bricks.

In the initial phase of a themed suite of bricks, students with advocacy experience in the area under consideration, such as Planetary Health, propose an outline, in which the bricks, learning objectives and content to cover to meet learners’ needs are identified. This outline is reviewed by the educator, an academic with expertise in the field, and finalized after asynchronous feedback between the students and the educator. ScholarRx’s curriculum manager reviews the outline to ensure the learning objectives and content organization suit the brick format. In the case of the Planetary Health suite, the feedback rounds during the outlining phase broadened the scope of the resources from seven bricks on climate change and health to 14 bricks on various Planetary Health topics, including air pollution, extreme weather events, and environmental justice.

Once the suite outline has been finalized, student authors are assigned to individual bricks, based on their stated preferences. Students apply to be authors via an online application form in which they share their academic information, such as graduation year, experience in research or medical writing, as well as their interest or experience in medical education. From these applicants, those with educational experience, such as leading peer-tutoring groups, are invited to write a section of the brick covering a specific learning objective. These assignments are reviewed and scored by two ScholarRx editorial team members, who evaluate the content quality and writing clarity. The candidates with the highest scores are then selected to develop the brick.

Each brick underwent three draft phases in which was reviewed by the educator and the developmental editor. At the second draft stage, the authors conduct a review on a different brick than the one they had developed (peer reviewer). The developmental editor may query the curriculum manager at this stage to resolve any issues related to the content organization or the pedagogical approach. At the final draft stage, artwork is developed according to the students’ instructions and placed in the bricks. The educator and curriculum manager signoff on the content before it is published.

## 3 Learning environment (setting, students, faculty); learning objectives; pedagogical format

The Planetary Health suite of 14 bricks aims to provide foundational knowledge on the topic, enabling future health professionals to recognize the environment as a determinant of health, identify the health impacts of a changing climate, biodiversity loss, and pollution, and advocate for strategies to reduce these impacts, such as practicing sustainable healthcare (Table 1):

- Introduction to Planetary Health
- Air Pollution
- Water Quality
- Climate Change and Health
- Extreme Heat
- Severe Weather Events
- Vector Ecology and Zoonoses
- Food and Water Security
- Mental Health and the Environment
- Climate Migration and Displacement
- Climate-Related Disaster Preparedness
- Climate Mitigation and Adaptation
- Environmental Justice
- Health Systems and the Environment

**Table 1:** The 14 Planetary Health bricks and the associated learning objectives.

Brick title	Learning objectives
Introduction to Planetary Health	Define planetary health and global environmental changes. Explain why the environment is a determinant of health. Identify planetary boundaries that are being exceeded. Explain the concept of sustainable development and doughnut economics.
Air Pollution	Define air pollution. Distinguish sources of air pollution. Describe the health effects associated with air pollution. Explain strategies to minimize the health effects of air pollution.
Water Quality	Define water quality, including the various harmful contaminants of fresh water. Explain the importance of clean water for human health. Identify the health effects of poor-quality water. Explain how to minimize the health effects of poor-quality water.
Climate Change and Health	Describe the dynamic interaction of elements in the climate system. Explain how human actions contribute to a changing climate. Identify climate-sensitive health risks. Define climate-resilient health systems.
Extreme Heat	Define extreme heat. Explain how extreme heat events are affecting health. Describe the clinical presentations associated with extreme heat. Discuss strategies to minimize the health effects of extreme heat.
Severe Weather Events	Distinguish types of severe weather events. Explain why severe weather events are increasing in frequency and intensity. Explain how natural disasters impact health. Explain how to minimize the health effects of severe weather.
Vector Ecology and Zoonoses	Define vector ecology and zoonoses. Explain the relationships between environment, host, and infectious agent. Identify climatic factors that contribute to infectious diseases. Explain the link between a changing climate and the increase in vectors and infectious diseases. Identify public health strategies to mitigate environmental risks for infectious diseases.
Food and Water Security	Define food and water security, including the state of food and water security worldwide. Explain how global environmental changes impact food and water security. List strategies to build climate-resilient food systems.
Mental Health and the Environment	Describe the relationship between mental health and the environment. Explain how environmental degradation impacts mental health. Identify strategies to address the impact of the planetary crisis on mental health.
Climate Migration and Displacement	Distinguish between the concepts of migration and displacement. Explain how environmental changes can lead to displacement. Identify the impact of climate migration and displacement on populations’ health. Recognize the importance of health interventions in the context of climate migration and displacement.
Climate-related Disaster Preparedness	Define climate-related disaster preparedness. Explain the impact of climate-related disasters on health and emergency care. List strategies to prepare for health emergencies during climate-related disasters. Explain the steps in the disaster management cycle.
Climate Mitigation and Adaptation	Define climate mitigation and adaptation. Define health co-benefits of climate mitigation and adaptation. Identify public health interventions promoting climate mitigation and adaptation. Explain climate resilience and ecosystem restoration
Environmental Justice	Define environmental justice. Explain why global environmental changes have a disproportionate impact on different regions. Identify the population groups most vulnerable to the impacts of global environmental changes. Explain strategies to reduce the impact of environmental changes.
Health Systems and the Environment	Describe the relationship between health systems and the environment. Describe the components of climate-resilient health systems. Explain the impacts of health systems on the environment. List strategies to promote environmentally sustainable health systems. Explain the role of health professions in promoting environmentally sustainable health systems.

The primary aim of the Planetary Health suite was to provide resources that can be used in their entirety by students and educators in the curriculum or as standalone learning resources that can be integrated into individual curriculum sessions. These bricks can be used as a self-directed learning resource, a pre-reading assignment before a face-to-face session, or integrated into a synchronous learning activity. The platform hosting the bricks currently collects data on the usage but does not evaluate or award a certificate for completion.

## 4 Results to date/assessment (processes and tools; data planned or already gathered)

**Figure 2:**
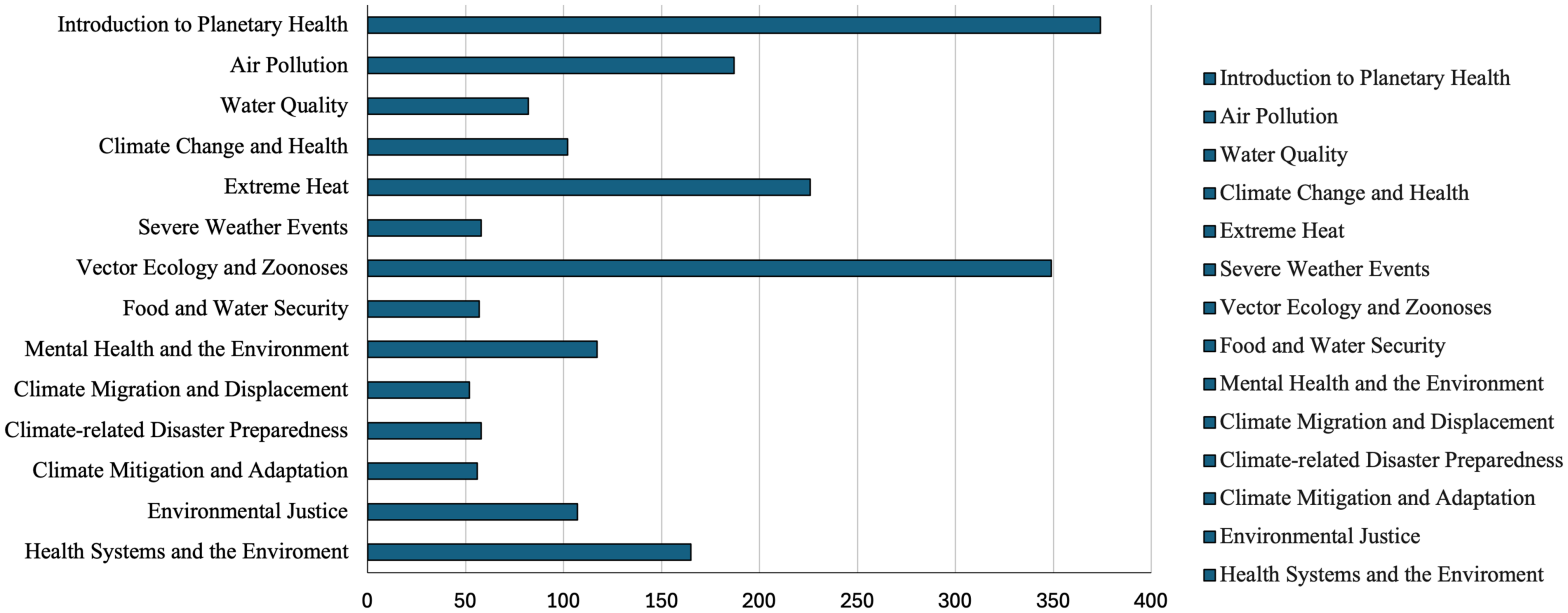
Number of brick views in the first 10 months after publication.

The bricks were published in June 2023. In the first 10 months after publication, the resources were viewed a total of 1,990 times, with *Introduction to Planetary Health*, *Air Pollution,* *Extreme Heat*, and *Vector Ecology and Zoonoses* garnering the most attention (Figure 2).

Following the release of the Planetary Health bricks on the ScholarRx platform, a webinar was held in October 2023 to introduce the Planetary Health suite to educators. The webinar was conducted as an interview, with the Curriculum Manager for the PH suite (CPR, author), posing key questions to the Planetary Health suite content expert (MM, author). An invitation for the webinar was sent to everyone on ScholarRx’s mailing list. Of the 78 registrants, 17 attended, with six providing feedback that included: “I hope to use them to educate peer educators,” “I have never used them before, but I am going to incorporate these in my curriculum now,” and “Fantastic guide for how to advocate for its implementation in vet[inerary] curricula.” All registrants received a link to the recording.

In 2023, MeSAGE ran two social media campaigns on Instagram promoting the Planetary Health suite. The content reached over 45,000 people, generated 2,000 interactions, and led more than 750 people exploring the collection.

During 2024, two online sessions were arranged to introduce students to the topic of Planetary Health and the suite of bricks. A session titled *“Advancing Planetary Health through Open Education”* was held during Open Education week in March 2024, and another session was held during the IFMSA’s European Regional Meeting in April 2024. The Planetary Health Bricks have also been added to OER Commons, a library of open educational resources.

MeSAGE plans to continue these implementation efforts by tracking usage data, delivering a set of synchronous peer-to-peer sessions based on bricks in the second half of 2024, and collecting direct feedback from educators on factors that support or hinder implementation.

## 5 Discussion on the practical implications, objectives, and lessons learned

There is an increasing call for HPE to include Planetary Health in the curriculum to ensure that graduates are prepared to practice sustainable healthcare and to be advocates for a cleaner, greener future for all. Health professions eeducators, who are often time-poor and may also not be Planetary Health experts, now have access to free open educational resources on which to draw. ScholarRx’s Planetary Health bricks offer educators free just-in-time and just-for-me resources that have been developed for students, by students and reviewed by an expert.

As a digital, open education resources published under a Creative Commons license, bricks can be shared freely allowing for easy dissemination and potential adoption by HPE education institutions worldwide. The digital format also allows for quicker updates and revisions, ensuring that the material remains current with the latest research and developments in Planetary Health. Due to its modularity and customizability, bricks can be easily incorporated in different programs and educators can tailor the content, depending on the context and learners’ needs.

While initial usage data is promising, a deeper understanding of the bricks effectiveness in meeting Planetary Health learning outcomes is required. Assessing the impact on student learning will be difficult, given the likely global usage of these resources. Surveys and focus groups with students and educators can provide information on the perceived learning gains, strengths and potential areas of improvement. There may also be opportunities for collaborations with educators who incorporate or integrate the bricks in their courses, so they can conduct pre- and post-assessment of learning objectives or track students’ learning and application of knowledge as it pertains to the topic of Planetary Health.

It is also important to note that developing innovative educational resources is an iterative process. As the resources are used, we aim to gather perspectives on potential improvements. A few weeks before the submission of this article, the platform upgraded its feedback features to prompt the users to share their perception of the learning experience. At the end of the brick, the users will see to buttons “thumbs up” and “thumbs down” and when they click on either, they can justify the feedback by selecting from a set of options (e.g., “clear and understandable,” “current and relevant information,” “difficult to understand,” “questionable or unreliable content”) or writing a comment. The expectation is that this data will inform subsequent revisions and updates to the content. New bricks addressing other aspects of Planetary Health should also be considered such as the health impacts MHPs and ‘forever’ chemicals, and biodiversity loss.

Finally, despite the merits of the current development process in terms of meaningful student engagement, there may be opportunities to further empower students. First, the student-educator collaboration can be strengthened by promoting synchronous sessions for alignment and feedback. Secondly, students can be encouraged to develop an activity to support the implementation of the brick they authored or the suite of bricks, which would encourage them to be educational activists and actively engage in their own education.

## 6 Acknowledgment of any conceptual, methodological, environmental, or material constraints

The topic of Planetary Health has not been systematically covered in health professions’ curriculum, thus specific references to guide the development of the resources were scarce.

## Data Availability

The original contributions presented in the study are included in the article/supplementary material, further inquiries can be directed to the corresponding author.
